# Socioeconomic inequalities in food insecurity and malnutrition among under-five children: within and between-group inequalities in Zimbabwe

**DOI:** 10.1186/s12889-020-09295-z

**Published:** 2020-08-04

**Authors:** Akim Tafadzwa Lukwa, Aggrey Siya, Karen Nelwin Zablon, James Mba Azam, Olufunke A. Alaba

**Affiliations:** 1 Health Economics Unit, School of Public Health and Family Medicine, Faculty of Health Sciences, University of Cape Town, Anzio Road, Observatory, Cape Town, 7925 South Africa; 2 College of Veterinary Medicine, Animal Resources and Biosecurity, Makerere University, P.O. Box 7062, Kampala, Uganda; 3 Centre for Invasion Biology, Department of Botany and Zoology, Stellenbosch University, Stellenbosch, South Africa; 4 National Institute for Medical Research, P.O Box 1462, Mwanza, Tanzania; 5 DSI-NRF Center of Excellence in Epidemiological Modelling and Analysis (SACEMA), Department of Mathematics, Stellenbosch University, Private Bag X1, Matieland, Stellenbosch, 7602 South Africa

**Keywords:** Food insecurity in children, Malnutrition in children, Under-five child health, Socioeconomic inequalities in children, Decomposing the Theil index

## Abstract

**Background:**

Food insecurity and malnutrition in children are pervasive public health concerns in Zimbabwe. Previous studies only identified determinants of food insecurity and malnutrition with very little efforts done in assessing related inequalities and decomposing the inequalities across household characteristics in Zimbabwe. This study explored socioeconomic inequalities trend in child health using regression decomposition approach to compare within and between group inequalities.

**Methods:**

The study used Demographic Health Survey (DHS) data sets of 2010\11 and 2015. Food insecurity in under-five children was determined based on the WHO dietary diversity score. Minimum dietary diversity was defined by a cut- off point of > 4 therefore, children with at least 3 of the 13 food groups were defined as food insecure. Malnutrition was assessed using weight for age (both acute and chronic under-nutrition) Z-scores. Children whose weight-for-age Z-score below minus two standard deviations (− 2 SD) from the median were considered malnourished. Concentration curves and indices were computed to understand if malnutrition was dominant among the poor or rich. The study used the Theil index and decomposed the index by population subgroups (place of residence and socioeconomic status).

**Results:**

Over the study period, malnutrition prevalence increased by 1.03 percentage points, while food insecurity prevalence decreased by 4.35 percentage points. Prevalence of malnutrition and food insecurity increased among poor rural children. Theil indices for nutrition status showed socioeconomic inequality gaps to have widened, while food security status socioeconomic inequality gaps contracted for the period under review.

**Conclusion:**

The study concluded that unequal distribution of household wealth and residence status play critical roles in driving socioeconomic inequalities in child food insecurity and malnutrition. Therefore, child food insecurity and malnutrition are greatly influenced by where a child lives (rural/urban) and parental wealth.

## Background

Malnutrition and food insecurity are major public health problems globally, and mostly dominant in low and middle income countries (LMICs) [1]. An estimate of 1 billion people are reported to be starved and malnourished [2]. About 45% of global deaths among children under five years were attributed to malnutrition [2]. Literature shows the burden of child malnutrition to be unequally distributed within regions and among countries [1–7].

**Fig. 1:**
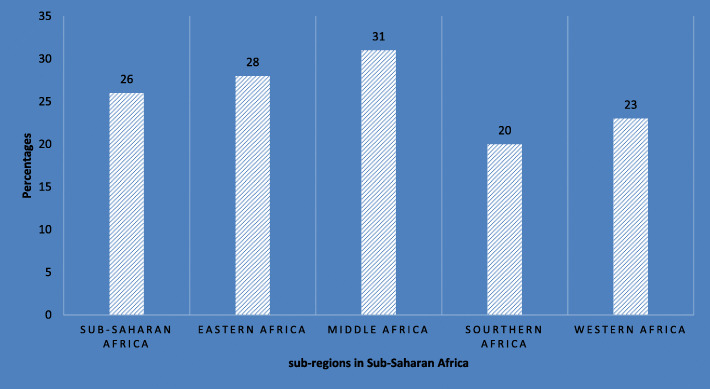
Prevalence of child food insecurity across sub-regions in Sub-Saharan Africa; Source: [9]

However, one in three children under the age of five is reported to be stunted, with middle and eastern Africa accounting for the highest proportions thus 31% and 28% respectively, while the lowest prevalence (20%) is observed in southern Africa [8] (Fig. 1). Figure 1 gives an overview of child food insecurity as of 2016 it can be deduced that middle Africa recorded the highest proportion 31% whilst southern Africa recorded the lowest.

An estimated one-third of globally malnourished children reside in sub-Saharan Africa (SSA) [7]. Stunting, severe wasting and intrauterine growth retardation are drivers of under-five mortality, accounting for about 3.1 million global deaths annually [2]. Health Economics literature reflects considerable evidence, arguing that nutrition and food security in children heavily relies on socioeconomic factors [1–3, 6, 7, 10–15]. Several studies have shown existence of socioeconomic inequalities in child malnutrition against child health determinants (age, sex and birth size of children) [2, 3, 8, 12, 15–19].

The food security framework is a multifaceted concept explaining interactions of food and poverty [20, 21]. Food insecurity can be defined as a state in which people do not possess physical and economic access to sufficient, safe and nutritious food, which satisfies their dietary needs [22]. Food security and nutritional statuses vary widely among children in households. Children from poor households are usually worse off than those from rich households [1]. Child malnutrition and food insecurity are common phenomena in rural inhabitants living in poverty [19].

Zimbabwe is part of Sub-Saharan Africa, situated to the south of Africa. Zimbabwe is a land locked country, whose Gross Domestic Product (GDP) comes mainly from agriculture and mining [23]. Agriculture is considered the back bone of Zimbabwe’s economy providing more than 70% of employment [24], with 2013–14 estimates reflecting a 13% contribution of agriculture to the country’s Gross Domestic Product [25].

However, Zimbabwe ranked 46/78 listed developing countries on the Hunger Index in 2013 [26]. On the hunger index ranking, Zimbabwe fell under the “Serious” category. Undernourishment was cited to be the major driving force for the latter stated position [24]. Poverty, and inadequate maternal and child care have been cited as the main driving factors of food insecurity and malnutrition in Zimbabwe [27].

The prevalence of stunting in Zimbabwe has been erratic since the mid-1980s’. However, post mid-1980 marginal declines in stunting were reported [28]. The rate of stunting among children accelerated from − 0.63% in 2012 to 0.20% in 2016, if this rate is maintained then in 2025, 760,000 children will be stunted [28].

Globally food insecurity and malnutrition are a cause for concern, hence their dominance on the global health agenda. The Sustainable Development Goal (SDG) 2.1 targets to: “End hunger and ensure access by all people, in particular the poor and vulnerable people, including infants, to safe, nutritious and sufficient food all year round by 2030” [29]. However, to achieve this there is need to investigate underlying causes of the problem.

Furthermore, most studies done in Zimbabwe on child food insecurity and malnutrition have explored mostly direction of association [20, 23, 30–33]. There is a need to show how certain socioeconomic attributes contribute to disparities in child food insecurity and malnutrition. This study explored socioeconomic health inequalities among children under-five in Zimbabwe. The study investigated socioeconomic differences of food insecurity and malnutrition, by assessing within and between group inequalities and compared two time periods 2010/11 and 2015.

## Methods

### Sources of data

Zimbabwe Demographic and Health Surveys (ZDHS) of 2010\11 and 2015 were used for analyses. Both data sets had population samples of 2714 and 2835 under-five children respectively, aged 0–59 months. Both 2010/11 and 2015, ZDHS samples were nationally representative composed of more than 11,000 households [34, 35]. The data sets gave representative information for most indicators in Zimbabwe for urban and rural areas [34–36].

The samples were representative of each of Zimbabwe’s ten provinces: Manicaland, Mashonaland Central, Mashonaland East, Mashonaland West, Matabeleland North, Matabeleland South, Midlands, Masvingo, Harare, and Bulawayo. The sampling frame for the 2002 and 2012 population census were used in both data sets [34–36].

### Outcome variables

The study assessed two indicators of child health in under-fives. The two indicators were endorsed by countries represented at the UN Statistical Commission to monitor target 2.1. Prevalence of undernourishment (malnutrition) and prevalence of severe food insecurity in the population were outcome variables [32].

Food insecurity among under-five children was determined using the WHO dietary diversity score, which is based on the Infant And Young Child Feeding (IYCF) practices. Dietary diversity is the number of different foods or food groups consumed over a given reference period [36].

For this study 13 food groups were considered, namely food from grains, food from tubers, eggs, meat, pumpkin & carrots, green leafy vegetables, vitamin A fruits, other fruits, liver & heart, fish, (beans, peas, lentils, nuts), other milk products and yogurt. The IYCF tool defines minimum dietary diversity as indicator for food security by a cut- off point of > 4 [37], in this study children with at least 3 of the 13 food groups were defined as food insecure. Children feeding responses in both surveys solemnly rely on the 24-h recall method, hence results on food security are prone to recall bias.

Malnutrition was assessed using the child anthropometric measure of weight-for-age. Weight-for-age is a composite index of height-for-age and weight-for-height hence takes account of both acute and chronic under-nutrition. Children whose weight-for-age z-score was below minus two standard deviations (− 2 SD) from the median were considered malnourished. Chi-square tests were used to assess the difference between food security status; nutritional status and socioeconomic classes, residence status, child age, and other background characteristics.

### Socioeconomic status

Socioeconomic status was adapted from the wealth index of households in the original surveys (ZDHS) [34, 35]. In both ZDHS’s, wealth index was reported as scores based on the number and kinds of consumer goods owned, ranging from a television to a bicycle or a car, plus housing characteristics such as source of drinking water, toilet facilities, and flooring materials [38]. The latter scores were derived using principal component analysis [38].

National wealth quintiles were compiled by assigning household scores, then each person was ranked in the household population by their score, and lastly the distribution was divided into five equal categories, each with 20% of the population in the original studies [34, 35]. For this study socioeconomic status was then re-categorised from 5 (poorer, poor, middle, richer, richest) groups into 3 groups thus poor, middle and rich (Table 1).

**Table 1:** Description of variables

**Child sex**	Recorded as; 0 male and 1 female
**Child age groups**	Recoded as: 0 neonate (1 day-1 month), 1 infant (1 month-24 months) and 2 young child (24 months–59 months)
**Mothers’ education**	Recoded as; 0 no education, 1 primary, tertiary educated
**Wealth index**	Recoded into 3 categories as; 0 Poor (poorer & poorest), 1 Middle, 2 Rich (richer & richest)
**Urban-rural residence**	Recoded 1 for urban and 2 for rural location

### Child age

The study focused on children under-five years of age (0-59 months). Children’s age was then recoded into 3 groups based on the South Africa’s department of health age definitions [39]. The 3 child age groups were defined as; Neonates (1 day-1 month), Infants (1 month-24 months) and Young children (24 months–59 months) (Table 1).

### Mother’s education

Zimbabwe’s education system is composed of 3 levels; primary education, secondary education and tertiary education [40]. The primary level is a seven-year cycle with an official entry age of six years running from Grade 1 to 7. However, prior to Grade 1 children are enrolled for early childhood education and care (preschool) for a year, but the latter is not formally considered as part of primary education.

Tertiary education in Zimbabwe covers all universities, technical colleges, polytechnic colleges, teacher’s training colleges and other vocational skills training canters [40]. Mother’s education was recoded into 3 categories thus; no education, primary and tertiary educated (Table 1).

### Erreygers normalised concentration index

The study used Erreygers normalised concentration indices [41], in determining socio-economic inequalities in child nutrition and food security. The study adopted the latter approach as the concentration index approach does not entirely measure inequalities in ordinal health variables [41, 42]. The latter index is expressed as a value of a health variable which would have been assigned to an individual as a function of a socioeconomic category to which the individual belongs [43].

Concentration index is a mathematical derivative of the concentration curve. On the concentration curve, the x-axis represents cumulative proportion of individuals by socioeconomic class starting with the lowest socioeconomic class (*poorest*) and ending with highest socioeconomic class (*richest*), while the y-axis is the cumulative total proportion of health in these individuals [44].

The Concentration curve identifies the existence of socioeconomic inequalities in health sector/outcome variables, and is only sensitive to relative inequality [44]. The bounds of this measure are − 1 and 1 with a negative (positive) value representing inequality favouring the worse-off (better-off).

Erreygers normalised index can be expressed algebraically as;

$$ I(h)=f\left({\mu}_n,n\right)\sum \limits_{i=1}^n{Z}_i{h}_i $$… (1).

Where;

Z_i_ represents number of individuals in a given population.

i denotes the socioeconomic rank of the individual ranging from the richest to the poorest.

h represents the health situation of the whole population.

### Theil index

The study used a generalized entropy measure known as the Theil index, mainly because of its decomposability [45]. The entropy measure is well-suited for estimating the contribution of different groups to total inequality [1]. Unlike other measures of inequalities like Gini index or The index of dissimilarity, generalised entropy class measures satisfy the five standard criteria for measuring inequalities including the attractive property of being easily decomposable by subgroups [41]. The Theil index is argued to be a measure of inequalities with distinctive properties, hence making it a powerful instrument in analysing patterns and dynamics of inequalities [46].

Generalised Entropy (GE) measures are cited to be based on the idea of divergence between probability distributions derived from information theory [45, 47–49]. Inequality decomposition was done by population subgroup to separate total inequality in the distribution into components of inequalities between the selected groups and the remaining within-group inequality. The study focused on decomposition by place of residence (rural/urban) and socioeconomic status (poor, middle and rich).

For this study we used the syntax ***ineqdec0***, which is a stripped-down version the syntax ***ineqdeco*** in Stata version 13.1. The study used ***ineqdec0*** syntax so as to include zeros and negative incomes in calculations. Theoretically, Theil index ranges from 0 to infinity, with 0 being a state of equal distribution and values greater than 0 representing increasing levels of inequality [50, 51]. Data analysis was done using Stata version 13.1 (Stata Corp, Texas, United States).

## Results

### Descriptive statistics

The study sample was composed of 2714 and 2835 under-five children for 2010/11 and 2015, respectively. Overall malnutrition prevalence and food insecurity prevalence for 2010/11 were, 3.73 and 78.29% while 2015 reported overall malnutrition prevalence and food insecurity prevalence of; 4.76 and 73.95%. Over the period under review, malnutrition prevalence increased by 1.03 percentage points (**p.p)** [2010/11(3.73%);2015(4.76%)] and food insecurity prevalence decreased by 4.34 percentage points (**p.p**).

For 2010/11; a greater proportion of malnourished children were poor, rural, boys whose mothers had attained at least secondary, while a greater proportion of food insecure children were poor, rural, girls whose mothers had at least attained secondary education (Table 2). While for 2015; both food insecure and malnourished children were reported to be poor, rural, girls whose mothers had at least attained secondary (Table 2). Residence status and socioeconomic status were statistically significant at 95% confidence interval (Table 2).

**Table 2:** Sample distribution and prevalence of malnutrition and food insecurity among children aged 0–59 months by Residence, Socioeconomic status, Mothers’ Education, Child age for Zimbabwe 2010/11 & 2015

Characteristics	Prevalence of malnutrition %		% Difference	Prevalence of food insecurity %		% Difference
	2010/11	2015		2010/11	2015	
*Child Sex*						
Male	54.42	49.01	−5.41	49.70	48.44	−1.26
Female	45.58	50.99	5.41	50.30	51.56	1.26
Chi-square	***0.00***	***0.34***		***0.08***	***0.01***	
*Household Wealth Index*						
Poor	46.56	56.16	9.6	51.75	53.75	2
Middle	20.19	20.33	0.14	19.03	18.41	−0.62
Rich	33.26	23.51	−9.75	29.22	27.84	−1.38
Chi-square	***0.00***	***0.00***		***0.00***	***0.01***	
*Mothers’ Education*						
No Education	1.47	1.34	−0.13	1.32	1.53	0.21
Primary	27.17	32.87	5.7	36.82	36.43	−0.39
Secondary	70.22	61.59	−8.63	59.62	58.96	−0.66
Tertiary	1.14	4.21	3.07	2.24	3.08	0.84
Chi-square	***0.34***	***0.39***		***0.61***	***0.35***	
*Child Age*						
Neonates	1.18	3.52	−2.34	5.03	7.12	−2.09
Infants	90.62	88.80	1.82	70.22	65.43	4.79
Young Children	8.20	7.65	0.55	24.75	27.44	−2.69
Chi-square	***1.47***	***1.86***		***1.79***	***1.89***	
*Residence*						
Urban	27.70	17.47	−10.23	21.06	20.56	−0.5
Rural	72.30	82.53	10.23	78.94	79.44	0.5
Chi-square	***0.00***	***0.00***		***0.00***	***0.00***	

### Concentration curves and indices

The Erreygers normalised concentration indices for nutrition status were all negative (pro-poor) for both time periods, translating to poor children less likely to be nutritious (Table 3). However, the Erreygers normalised concentration index for 2010/11 was not statistically significant at 95% confidence interval. Erreygers normalised concentration indices for food security status were positive (pro-rich) for both time periods meaning children from wealthy households were more likely to be food secure (Table 3). For both time periods, socioeconomic inequalities appear to be widening for child nutrition and food security status, this is reflected by the increasing Erreygers normalised concentration indices.

**Table 3:** Erreygers Normalised Concentration indices for nutrition status and food security status

***Food Security Status***			
***Period***	***Erreygers Normalised Concentration Index***	***Standard Error***	***P-value***
***2010–11***	0.1610	0.0181	0.000
***2015***	0.2093	0.0191	0.000
***Absolute difference***	0.0483 ↑		
***Nutritional Status***			
***Period***	***Erreygers Normalised Concentration Index***	***Standard Error***	***P-value***
***2010–11***	−0.0028	0.0084	0.737
***2015***	−0.0264	0.0094	0.005
***Absolute difference***	0.0236 ↑		

**Fig. 2:**
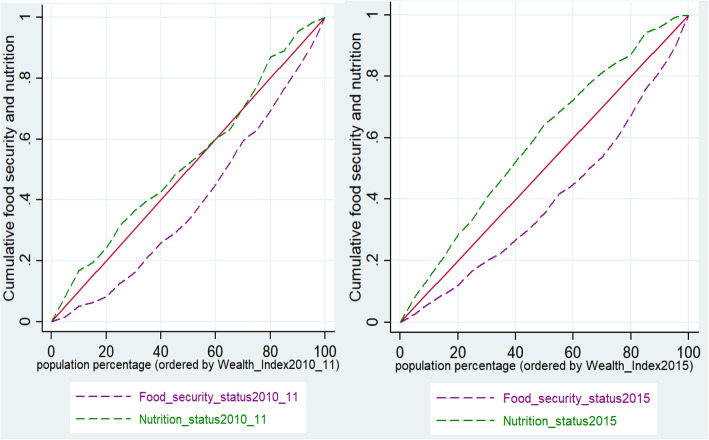
a and b; Concentration curves of food security and malnutrition for 2010/11 and 2015.

Computed concentration curves for malnutrition status for both time periods are presented in Fig. 2a;b. The curves showed that children from low socioeconomic classes were less likely to be nutritious. However, for food security status in 2015, the curve showed that children from wealthy households were more likely to be food secure (Fig. 2b). For 2010/11, the concentration curve crossed the 45-degree line of equality at some points (Fig. 2a), which prompted the computation of the dominance test.

Test of dominance between nutrition_status2010_11 concentration curve and the 45-degree line showed non-dominance of the concentration curve (Table 4). Cumulative quintile shares for Nutrition_status_2010_11 showed that the poorest 20% children accounted for only 20.7% of nutritious children (Table 4). *P*-values of household wealth share indicated that nutritional status shares were not significantly different from the nutritional status shares for all quintiles (Table 4).

**Table 4:** Test of dominance between Nutrition_status2010_11 concentration curve and 45-degree equality line

***Variable***		***Significance level***	***Number points***	
Nutrition_status2010_11		5%	19	
**Non-dominance**				
***Cumulative quintile shares for Nutrition_status_2010_11 reported with the output***				
***Quantile***	***Standard Error***	***Cumulative share*** ***(%)***	***Difference from population share*** ***p-value***	***Difference from household wealth share*** ***p-value***
***Q***^***20***^	3.973	20.7	0.869	0.000
***Q***^***40***^	4.759	38.5%	0.754	0.000
***Q***^***60***^	4.877	55.1%	0.317	0.000
***Q***^***80***^	3.320	88.1%	0.014	0.000

### Decomposition analysis

The study focused on two household characteristics in assessing health inequalities: location of the household and socioeconomic status/class. Socioeconomic inequalities among regions and socioeconomic classes within a country can be attributed as the driving force inducing uneven progress of economic development across regions [52].

### Decomposition by residence

The bigger proportion of children resided in the rural areas and also accounted for the greatest hosehold wealth share (Table 5). There was insignificant variance between household wealth share and population share, thus making place of residence (urban & rural) comparable (Table 5).

**Table 5:** Population share and household wealth share of subgroups for food security and nutritional status distinguished by residence; 2010/11 & 2015

**Food Security**								
**Region**	**2010/11**				**2015**			
	**Population share**	**Mean**	**Relative mean**	**Wealth share**	**Population share**	**Mean**	**Relative mean**	**Wealth share**
**Urban**	0.2415	0.30	1.47	0.3555	0.3198	0.37	1.45	0.4629
**Rural**	0.7585	0.16	0.85	0.6445	0.6802	0.20	0.79	0.5371
**Nutritional Status**								
**Region**	**2010/11**				**2015**			
	**Population share**	**Mean**	**Relative mean**	**Wealth share**	**Population share**	**Mean**	**Relative mean**	**Wealth share**
**Urban**	0.2415	0.31	1.00	0.2407	0.3198	0.03	0.66	0.2114
**Rural**	0.7585	0.04	1.00	0.7593	0.6802	0.05	1.16	0.7886

Table 6 showed contracting socioeconomic inequality gaps for food security for both time periods between rural and urban children. However, socioeconomic inequalities in food insecurity were more dominant in the rural areas as it recorded Theil indices higher than in the urban. For nutritional status, socioeconomic inequalities appear to have widened in the urban areas while in the rural areas the gaps contracted. Theil indices in the urban increased while in the rural areas the indices decreased (Table 6).

**Table 6:** Theil indices for subgroups for food security status and nutritional status distinguished by residence and socioeconomic classes

	**Theil index decomposed by residence**					
	**Food security status**			**Nutritional status**		
**Region**	**2010/11**	**2015**		**2010/11**	**2015**	
	**Theil index [*GE (2)*]**	**Theil index [*GE (2)*]**	**Absolute difference**	**Theil index [*GE (2)*]**	**Theil index [*GE (2)*]**	**Absolute difference**
**Urban**	1.1465	0.8364	−0.3101	12.0385	16.1539	4.1154
**Rural**	2.3524	1.9495	−0.4029	11.9878	9.0000	−2.9878
	**Theil index decomposed by socioeconomic status**					
**Socio- economic class**	**Food security status**			**Nutritional status**		
	**2010/11**	**2015**		**2010/11**	**2015**	
	**Theil index [*GE (2)*]**	**Theil index [*GE (2)*]**	**Absolute difference**	**Theil index [*GE (2)*]**	**Theil index [*GE (2)*]**	**Absolute difference**
**Poor**	3.1453	2.2854	−0.8599	11.3636	8.3134	−3.0502
**Middle**	1.4222	1.6900	0.2678	14.7647	9.4546	−5.3101
**Rich**	1.3025	0.9034	−0.3991	11.6667	15.5147	3.8480

Decomposed results of food security by residence for 2010/11 showed about 1.6% of the socioeconomic inequalities were explained by the child’s residence status (Table 7). More than 98% of socioeconomic inequalities were explained by within group household wealth variances (Table 7). Decomposed results for 2015, showed that 2.5% of socioeconomic inequalities were explained by the child’s residence status, while more than 97% were explained by within group household wealth variances (Table 7).

**Table 7:** Decomposition of the Theil indices by residence and socioeconomic classes for food security status and nutritional status

	**Overall, within and between group inequalities by residence**							
**Region**	**Food security status**				**Nutritional status**			
	**2010/11**		**2015**		**2010/11**		**2015**	
	**Theil index [*GE (2)*]**	**Contribution**	**Theil index [*GE (2)*]**	**Contribution**	**Theil index [*GE (2)*]**	**Contribution**	**Theil index [*GE (2)*]**	**Contribution**
**Overall**	1.9237	100%	1.4343	100%	12.0000	100%	10.5081	100%
**Within-Group**	1.8882	98.4%	1.3872	97.5%	12.0000	96.4%	10.4811	92.7%
**Between Group**	0.0355	1.6%	0.0471	2.5%	0.0000	3.6%	0.0270	7.3%
	**Overall, within and between group inequalities by socioeconomic classes**							
**Region**	**Food security status**				**Nutritional status**			
	**2010/11**		**2015**		**2010/11**		**2015**	
	**Theil index [*GE (2)*]**	**Contribution**	**Theil index [*GE (2)*]**	**Contribution**	**Theil index [*GE (2)*]**	**Contribution**	**Theil index [*GE (2)*]**	**Contribution**
**Overall**	1.9237	100%	1.4343	100%	12.0000	100%	10.5081	100%
**Within-Group**	1.8708	97.2%	1.3840	95.7%	11.9960	94.3	10.4741	96.4%
**Between Group**	0.0530	2.8%	0.0502	4.3%	0.0040	5.7%	0.0341	3.6%

For nutrition in 2010/11 over 96% of socioeconomic inequalities were explained by within group household wealth variability and about 4% explained by child’s residence status (Table 7). While in 2015 within group household wealth variations explained over 92% of socioeconomic inequalities, with about 7% explained by child’s residence status (Table 7). Therefore, food security within group household wealth variations explained 97.5–98.4% of socioeconomic inequalities, while where the child resided explained 1.6–2.5% of socioeconomic inequalities.

### Decomposition by socio-economics status

Table 6 showed contracting socioeconomic inequality gaps among the poor for food security status and nutritional status for the period under review. However, for the wealthy class socioeconomic inequality gaps appeared to have contracted for food security status and widened for nutritional status (Table 6). Table 7 results showed that when decomposed by socioeconomic status for food security 95.7–97.2% of socioeconomic inequalities were explained by within group household wealth variations. About 2.8–4.3% socioeconomic inequalities were explained by the socioeconomic group to which the child belonged.

For nutrition 94.3–96.4% socioeconomic inequalities were explained by within group household wealth variations and 3.6–5.7% socioeconomic inequalities were explained by the socioeconomic class to which the child belonged (Table 7). For food security in both time periods, the lowest socioeconomic class accounted for the biggest population share and the second least proportion of wealth share (Table 8).

**Table 8:** Population share and household wealth share of subgroups for food security and nutrition distinguished by socio-economic classes; 2010/11 & 2015

**Socioeconomic class**								
**Food Security Status**	**2010/11**				**2015**			
	**Population share**	**Mean**	**Relative mean**	**Wealth share**	**Population share**	**Mean**	**Relative mean**	**Wealth share**
**Poor**	0.4833	0.14	0.67	0.3214	0.4361	0.18	0.69	0.3029
**Middle**	0.1922	0.26	1.26	0.2424	0.1617	0.23	0.88	0.1429
**Rich**	0.3244	0.28	1.34	0.4363	0.4021	0.36	1.34	0.5543
**Nutritional Status**								
	**2010/11**				**2015**			
	**Population share**	**Mean**	**Relative mean**	**Wealth share**	**Population share**	**Mean**	**Relative mean**	**Wealth share**
**Poor**	0.4833	0.04	1.05	0.5093	0.4361	0.06	0.55	0.5447
**Middle**	0.1922	0.03	0.82	0.1574	0.1617	0.05	0.18	0.1789
**Rich**	0.3244	0.04	1.03	0.3333	0.4021	0.03	0.28	0.2764

## Discussion

Socioeconomic status (wealth index) and residence status were significant predictors of food insecurity and malnutrition in our study. Availability and variety of food in the household heavily relies on economic status [15]. In our study rural children from poor households accounted for the greatest proportion of food insecure and malnourished children. The latter findings were consistent with arguments observed in literature [1, 15, 16, 19, 37, 53]. As child food insecurity and malnutrition are widespread phenomenon’s, dominant in rural areas, where most of the inhabitants live in poverty [7].

This study reported a decrease in food insecurity prevalence. This is in contrary to what was observed in Ethiopia, as generally under-fives of this country did not meet the minimum required dietary diversity [15]. However, our results reported an increase in malnutrition among poor households, thus reflecting widening of the poor-rich divide in Zimbabwe. Therefore, this argues that even though children were getting food it was not nutritious. This was consistent with findings from other African countries [1, 2, 7, 12, 15, 19, 53]. The existence of disparities in malnutrition and food insecurity in Zimbabwe within subpopulations, disproportionately affected the poor residing in the rural areas mostly. Considering the impact of poverty on malnutrition, the health sector is essential in mitigating such distress through provision of basic health amenities.

Literature has a reasonable body of evidence linking maternal knowledge to Infant And Young Child Feeding (IYCF) (food insecurity) and quality of children’s diet (malnutrition) [2, 6, 7, 11, 17, 18, 26, 28, 32, 52–54]. This was not the same in our study as mother’s education was not statistically different at 95% confidence interval. However, food insecurity and malnutrition were most prevalent among women who attained at least secondary education. A study done in Nigeria reported child’s age as one of the largest contributors to socioeconomic inequalities to undernutrition for children under-five years [1]. However, this was not the same with our study, as child age was not statistically different at 95% confidence interval.

The concentration indices estimate for nutritional status were all negative and corresponded with the concentration curves for both time periods, reflecting that poor children were disproportionately affected by malnutrition in Zimbabwe. The study findings concur with what was observed in Ghana, were stunting and wasting affected the poor disproportionately [12]. This reflects worsening of health conditions among rural children thus, increasing the urban-rural gaps in child health.

Decomposed results of this study showed within group household wealth variations to be strong indicators explaining socioeconomic inequalities, the latter findings also concur with results of other studies [12, 52]. More recently, a study used Shapley decomposition to estimate the relative contributions of circumstances and analysed patterns of inequalities in health relative to nutrition outcomes among children under five in Tunisia [18]. Findings of the Tunisia study revealed reasonably and low levels of inequalities in access to all basic healthcare services and nutrition except access to improved water and sanitation. However, the study reported parents’ education, wealth and place of residence as key determinants causing the low inequalities [18]. In our study wealth and place of residence were determinants driving the socioeconomic inequalities.

The Ghana findings, reported parental wealth and place of residence as key factors influencing socioeconomic inequalities in child health [18]. This was also true for our findings, when we decomposed the inequalities by socioeconomic status and place of residence. The decomposed results showed that food insecurity should simultaneously tackled with malnutrition, so as to allow for the addressing of different nutritional challenges [14]. Key message to bear in mind is that malnutrition and food insecurity in children are not merely food and dietary problems in Zimbabwe. The two child health problems are shaped by broader societal and economic context based on where children reside [14, 55].

This research had some limitations. Firstly, as the study is a cross-sectional design, it was impossible to establish causal relationships between food insecurity & malnutrition and socioeconomic variables. Secondly, although weight-for-age has been recommended as the anthropometric criteria for diagnosis of acute undernutrition among children under five 5 years, there is minimal data describing its reliability [56]. Therefore, malnutrition interpretation might be limited for this age group. Thirdly, this study did not directly measure household income. Therefore, using wealth index as a proxy measure might have affected the impact of child health determinants on food insecurity and malnutrition. Lastly, appetite and illness are variables that influence dietary intake in children [15], however such variables were not accounted for in the study.

Despite the highlighted study limitations, this study also had some merits. First, demographic health surveys are population-based surveys with large samples, hence the samples were nationally representative of Zimbabwe. Lastly, the study compared within and between group socioeconomic inequalities, which is important policy evidence in public health planning in relation to food insecurity and malnutrition in children.

### Policy recommendations

Considering the high inequality of opportunity among rural poor children, special attention should be paid to equal distribution of public services across regions to enhance fair chances for child health. Adequate child nutrition is important for proper cognitive and physical development of children [1]. Curbing malnutrition in children is crucial, as malnutrition in children has been associated with long-term costs, for instance adverse health conditions with consequent effects on the labour force in future [55]. The latter shows economic gains which can be realised by minimising child malnutrition.

An important way of bridging the rural–urban gap with respect to child malnutrition will be to strengthen and make use of close-to-client health care arrangements [57]. This implies use of Community- based Health Planning and Services; Supplementary Feeding and Health and Nutrition Education Programmes in deprived communities, mostly rural communities through community health volunteers. As already Zimbabwe has the village health worker system in place, adapting similar policies across health programs should be doable, considering that the infrastructure already exists.

A study done in Ethiopia reported that mothers who had received Infant And Young Child Feeding (IYCF) information during antenatal care (ANC) and postnatal care (PNC) check-ups were likely to offer food to their young children from four or more food groups [15]. This can be an important tool to adopt, as it reaches a wider audience (pregnant women and mothers).

## Conclusion

Therefore, this study concludes that unequal distribution of household wealth and residence status play critical roles in driving socioeconomic inequalities in child food insecurity and malnutrition. Therefore, child food insecurity and malnutrition are heavily influenced by where a child lives (rural/urban) and parental wealth. In a nutshell, intuitively the inferior socioeconomic status restricts the availability and variety of food in a household.

## Data Availability

All data sets are publicly available on the Demographic Health Survey website and can be accessed upon request from the Demographic Health Survey team.

## Abbreviations

**ANC**: Antenatal Care
**DHS**: Demographic Health Survey
**GDP**: Gross Domestic Product
**GE**: Generalised entropy (GE)
**IYCF**: Infant and Young Child Feeding
**SD**: Standard Deviation
**SDG**: Sustainable Development Goals
**PNC**: Postnatal Care
**UN**: United Nations
**WFP**: World Food Programme
**WHO**: World Health Organisation
**ZimVac**: Zimbabwe Vulnerability Assessment Committee
